# Use of Precision-Cut Lung Slices as an *Ex Vivo* Tool for Evaluating Viruses and Viral Vectors for Gene and Oncolytic Therapy

**DOI:** 10.1016/j.omtm.2018.07.010

**Published:** 2018-08-04

**Authors:** María C. Rosales Gerpe, Jacob P. van Vloten, Lisa A. Santry, Jondavid de Jong, Robert C. Mould, Adrian Pelin, John C. Bell, Byram W. Bridle, Sarah K. Wootton

**Affiliations:** 1 Department of Pathobiology, University of Guelph, Guelph, ON N1G 2W1, Canada; 2 Ottawa Hospital Research Institute, Centre for Innovative Cancer Research, Ottawa, ON K1H 8L6, Canada; 3 Department of Biochemistry, Microbiology and Immunology, University of Ottawa, Ottawa, ON K1H 8M5, Canada

## Abstract

Organotypic slice cultures recapitulate many features of an intact organ, including cellular architecture, microenvironment, and polarity, making them an ideal tool for the *ex vivo* study of viruses and viral vectors. Here, we describe a procedure for generating precision-cut ovine and murine tissue slices from agarose-perfused normal and murine melanoma tumor-bearing lungs. Furthermore, we demonstrate that these precision-cut lung slices can be maintained up to 1 month and can be used for a range of applications, which include characterizing the tissue tropism of viruses that cannot be propagated in cell monolayers, evaluating the transducing properties of gene therapy vectors, and, finally, investigating the tumor specificity of oncolytic viruses. Our results suggest that *ex vivo* lung slices are an ideal platform for studying the tissue specificity and cancer cell selectivity of gene therapy vectors and oncolytic viruses prior to *in vivo* studies, providing justification for pre-clinical work.

## Graphical Abstract

**Figure:**
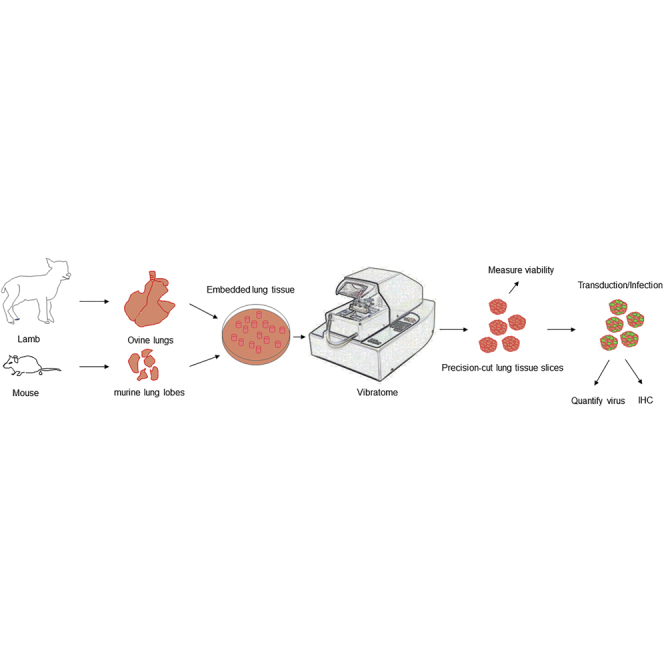

## Introduction

The preparation of tissue microslices for *ex vivo* experimentation is a technique that produces precision-cut organotypic slices with the use of a vibratome or tissue slicer that operates with a vibrating blade. The use of a vibrating blade allows for greater accuracy and reproducibility when generating precision-cut lung slices (PCLSs).^1^ Compared to *in vivo* work, tissue slices are cost-effective and less time-consuming while still possessing many of the attributes of the intact organ.^1^, ^2^, ^3^ In fact, PCLSs have been utilized in the study of lung anatomy because they maintain the structural framework at both the tissue and cellular level while also retaining all relevant cell types.^1^, ^2^, ^4^, ^5^, ^6^, ^7^ PCLSs are often utilized in toxicological and anatomical studies regarding contractility in relation to asthma and other respiratory illnesses, such as emphysema.^1^, ^5^ Recently, lung slices have been used in immunological studies, where they have been shown to retain their immune cell populations and functions, which mimic that of the organ.^4^, ^8^, ^9^, ^10^

With regard to infectious diseases, lung slices have been used as a model system to study respiratory pathogens, including adenoviruses, influenza viruses, respiratory syncytial virus, and the bacterium *Clamydophila pneumoniae*, ultimately contributing to our understanding of the pathogenesis and replication cycles of these infectious agents.^8^, ^9^, ^10^, ^11^, ^12^ Additionally, lung slices have been used to evaluate the transducing activity of various viral vectors.^13^, ^14^, ^15^, ^16^, ^17^, ^18^, ^19^ For example, in a study evaluating lentiviral vectors (LVs) pseudotyped with the Ebola virus (EBOV) glycoprotein (G) as a potential gene delivery vector,^14^, ^15^ the EBOV G-bearing LVs did not infect cell monolayers as efficiently as vesicular stomatitis virus (VSVG)-G-pseudotyped LVs; however, the EBOV G-pseudotyped LV was more efficient at transducing cells in three-dimensional culture than VSV-G LV.^15^ Finally, PCLSs can be used as a platform to study viruses, such as Jaagsiekte sheep retrovirus (JSRV), that are unable to replicate in cell monolayers due to their requirement for cell membrane polarity.^14^, ^15^, ^20^, ^21^, ^22^, ^23^, ^24^, ^25^, ^26^, ^27^, ^28^, ^29^ Testing viruses in tissue slices would also expedite and help to refine virus-driven gene therapy and oncolytic virus (OV) studies, which ultimately require the use of *in vivo* models to test their efficacy. Thus, the use of lung slices that preserve many of the *in vivo* conditions can be used to predict gene and oncolytic therapy outcomes in animal models.

To this end, we present a detailed protocol for generating lung tissue slices from both small (mice) and large (sheep) animals and from normal and tumor-bearing tissue. Generating slices from the latter preserves the tumor macro- and microenvironment that can never be recapitulated in conventional cellular monolayers or three-dimensional cultures and allows tumor selectivity of an OV to be evaluated relative to adjacent normal tissue. We show that this protocol can be used to successfully propagate viruses with cell polarity issues (JSRV), allow for efficient transduction with gene therapy vectors (VSV-G-pseudotyed LV and adenovirus GFP [Ad-GFP]), and support infection with OVs (Maraba [MG1-enhanced GFP (eGFP)], vaccinia [VACV-GFP], and Newcastle disease virus [NDV-GFP]). Notably, this protocol could readily be adapted to a broad array of viruses and tissue types.

## Materials

### Reagents

• Low melting point agarose ultrapure (Fisher Scientific, 16520100)
• 1X Hank’s buffered salt solution (HBSS) with Ca^2+^/Mg^2+^ (Fisher Scientific, SH3026802)
• Industrial-strength glue (Loctite Super Glue Ultra Gel Control)
• PBS without calcium/magnesium/phenol red (Fisher Scientific, SH3037803)
• DMEM high glucose with L-glutamine (Fisher Scientific, SH30022FS)
• Recombinant human hepatocyte growth factor (Cedarlane, 100-39-10UG(PE))
• Recombinant human keratinocyte factor (GIBCO Life Technologies, PHG0094)
• L-glutamine (Fisher Scientific, SH3003402)
• Penicillin/streptomycin (Fisher Scientific, SV30010)
• Amphotericin B (Fisher Scientific, SV3007801)
• Gentamycin (Fisher Scientific, 15710064)
• Dexamethasone (Sigma-Aldrich, D4902-25MG)
• 8-Bromo-adenosine 3′,5′-cyclic monophosphate (Sigma-Aldrich, B5386-5MG)
• 3-Isobutyl-1-methylxanthine (Sigma-Aldrich, I5879-100MG)
• Hexadimethrine bromide (Polybrene) (Sigma-Aldrich, H9268-5G)
• Fetal bovine serum (Fisher Scientific, 12483020)
• 10% Formalin (Fisher Scientific, SF100-4)
• Ethanol (Fisher Scientific, A962F-1GAL) to make 70% ethanol
• Isopropanol (Fisher Scientific, BP2618-4)
• Xylene (Fisher Scientific, HC7001GAL)
• Hematoxylin (Fisher Scientific, SH26-500D)
• Superfrost Plus slides (ThermoScientific, 22-037-246)
• C57BL/6 Mice (Charles River)
• Specific-pathogen-free (SPF) Cornell Star Sheep (neonates: 6-month-old lambs)
• Live/dead viability dye (Life Technologies, LSL7013)
• Resazurin dye (Sigma-Aldrich, R7017-5G)

### Equipment

• Styrofoam box lids, saran wrap, and wooden skewers
• 8 mm tissue puncher (TedPella Biopunch, 15111-80)
• 50 mL centrifuge tubes (Fisher Scientific, 14955240)
• Petri dishes 100 × 15 mm (Fisher Scientific, FB0875712)
• 48-well plates (VWR, 10062-898)
• 96-well plates (VWR, 10062-900)
• Ted Pella Microslicer DTK-3000W (Vibratome)
• Nutating mixer fixed speed 120 V (Fisher Scientific, 88861041)
• 5 and 20 mL syringes
• 18G needles
• Fine-point forceps
• Scalpel
• Surgical scissors
• Clamps (hemostats)
• p1000 Gilson pipette
• p1000 pipette filter tips
• 10 mL serological pipettes
• KimWipes (Sigma-Aldrich, Z188956)
• CO_2_ incubator
• Fluorescent microscope
• Foam biopsy sponges 25 × 31 mm (ThermoFisher Scientific, 8453)
• Micromesh loose biopsy cassettes (Thermofisher, B1000735WH)
• Microscope cover glass (Fisher Scientific, 12-545-C 22X40-1)
• Superfrost Plus microscope slides (Fisher Scientific, 12-550-15)
• Dakocytomation pen (Dakocytomation, S2002)
• ThermoScientific Excelsior ES tissue processor
• ThermoScientific HistoStar tissue embedding station
• ThermoScientific Finesse ME+ microtome

### Reagent Setup

#### Low Melting Point Agarose Gel

Prepare 2% (w/v) low melting point agarose (LMPa) in 1X HBSS 1 hr prior to perfusion of the lungs by adding 4 g of LMPa to 200 mL of 1X HBSS and allowing it to dissolve by heating via a microwave by pulsing every 2 min, or until boiling is observed, for approximately 6 min until the LMPa has solubilized. Once the LMPa has solubilized, distribute the 200 mL among four 50 mL centrifuge tubes and place in a water bath pre-heated to 42°C to avoid solidification until ready to perfuse.

#### Wash and Maintenance Media Solutions

Please see Table 1 for recipes on how to make the appropriate medium. Make sure to have these solutions ready prior to cutting.

**Table 1:** Media Conditions for Lung Slice Maintenance and Use

Media	Reagents
Lung slice wash medium (LSWM)	50 U/mL penicillin/streptomycin 0.2 μg/mL gentamycin 1.25 μg/mL amphotericin 10 μM 8-bromo-adenosine 3′,5′-cyclic monophosphate 100 μM 3-isobutyl-1-methylxanthine 100 nM dexamethasone DMEM with high glucose
Lung slice maintenance medium for infection with VSV-G LV (LSMM-A)	reagents in LSWM and 10% FBS; 2 mM L-glutamine
Lung slice maintenance medium for infection with MG1-eGFP, VACV-GFP, NDV-GFP, and Ad-GFP (LSMM-B)	reagents in LSMM-A and 10 ng/mL recombinant human keratinocyte factor
Lung slice maintenance medium for infection with JSRV (LSMM-C)	reagents in LSMM-B and 5 ng/mL recombinant human hepatocyte factor

Lung slice wash and maintenance media composition. Four types of medium were used to treat the ovine and murine lung slices. A lung slice wash medium (LSWM) was used for both types of lung tissue slices to prevent contamination. Three different types of lung slice maintenance medium (LSMM) were used. Both the LSWM and LSMM contained the following antibiotics: penicillin, streptomycin, gentamycin, and amphotericin. LSMM-A contained the bare minimum standard media reagents such as 10% FBS and 1% L-glutamine and was used to culture the VSV-G GFP LV-transduced ovine lung slices. LSMM-B contained the same reagents as LSMM-A in addition to the recombinant epithelial growth factor. LSMM-B was used to culture murine lung tumor slices infected with MG1-eGFP, VACV-GFP, NDV-GFP, and Ad-GFP. Finally, LSMM-C was composed of the same reagents as LSMM-B but also contained a human recombinant hepatocyte factor required for propagation of JSRV in sheep lung slices.

#### Medium and Polybrene

For experiments involving LVs, use a final concentration of 8 μg/mL of polybrene (stock: 8 mg/mL; 1 μL polybrene to 1 mL medium) to aid in transduction.^30^ This can be prepared the day of transduction and infection with LV or JSRV, respectively.

### Equipment Setup

#### Perfusion of Ovine Lungs

**Figure 1:**
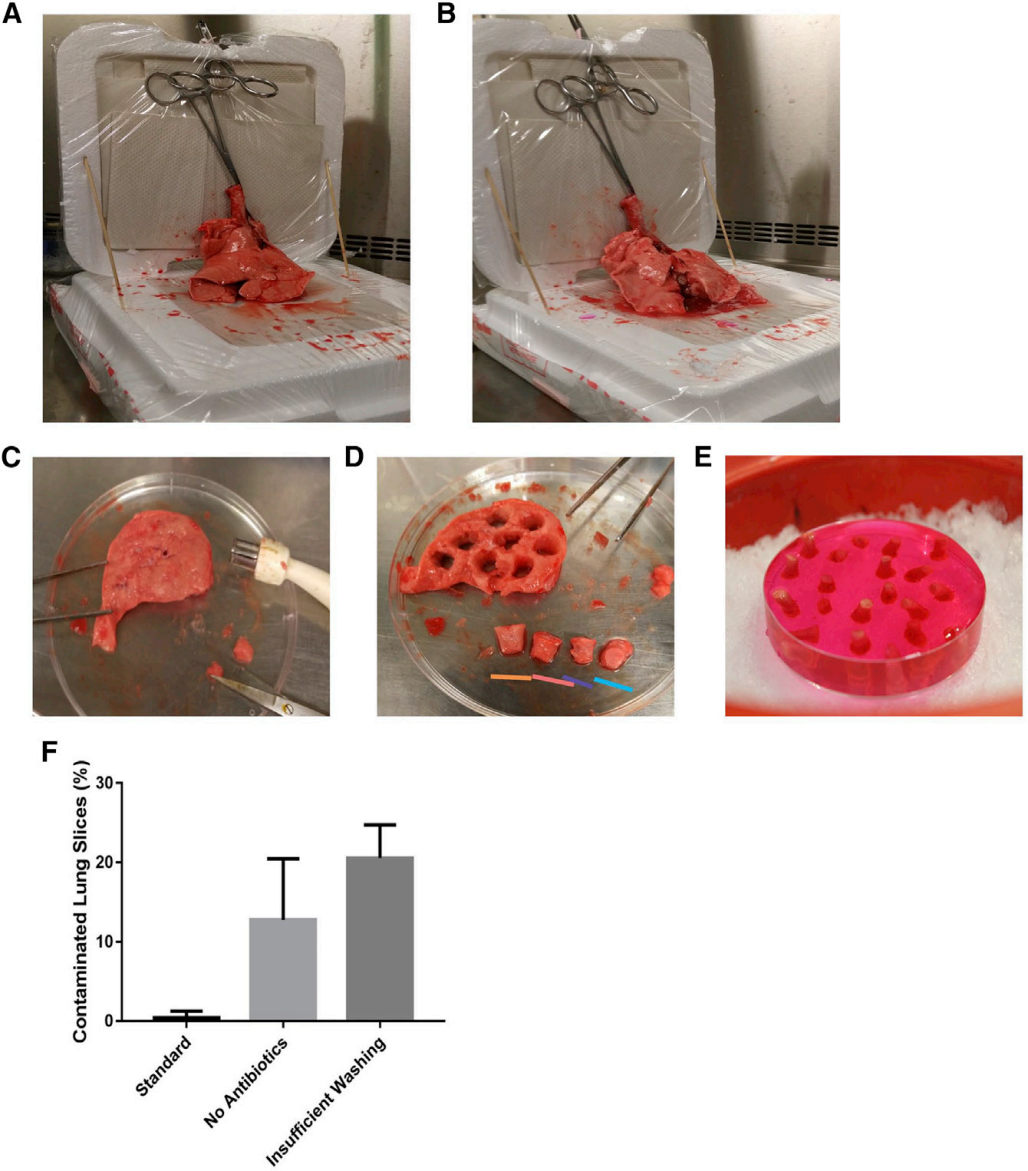
Setup for the Perfusion of Sheep Lungs (A) Two styrofoam box lids are lined with paper towels and enclosed with plastic wrap prior to being positioned 90° to each other with the use of wooden skewers. Clamps (hemostats) are then attached to the trachea and used to position the lungs upright. Another set of hemostats is put through the clamps holding the lungs and adhered to the vertical styrofoam cover. The initial state of the sheep lungs is characterized by the lobes appearing deflated and folded onto themselves (A); however, upon perfusion (B), they no longer fold and appear engorged. (C) A section approximately 2 mm thick is generated using a scalpel and then (D) an 8 mm puncher is used to core the slice to generate 8 mm-wide 2 mm-thick cores. The cores may vary in perfusion level, as the lungs will be differentially perfused throughout. The colored bars under the lung cores denotes the difference in perfusion level that can be observed in one section, with the orange bar being the highest, leaving the core spongy, and the light blue bar showing a flat section, lacking perfusion. The appropriate lung cores (pink bar or second from the left) can then be moved onto a Petri dish and embedded within LMPa (E). (F) The use of antibiotics extends culturing of sheep lung slices. Greater survival was observed when lung slices were cultured over a period of 1 to 4 weeks throughout 6 months with or without antibiotics. Those maintained in media with antibiotics were also subjected to 5 washes prior to culturing. Error bars represent SEM.

Set up a platform to maintain lungs in an upright position using styrofoam lids for insulation. See Figure 1.

#### Vibratome Settings

Remove the buffer tray platform and add ice to the reservoir. Turn on the lamp and set the blade settings to 100% frequency at a speed of 8 μm/s. Set the tissue cutting settings to 300 μm thickness, and a blade traveling range of 20–40 mm, depending on the number of blocks in the platform.

#### ThermoScientific Excelsior ES Tissue Processor Settings

Use the following program: six 23 s cycles of exposure to alcohols (70%, 85%, 90%, 95%, and 2 cycles of 100% isopropanol) at 30°C, three 23 s cycles in xylenes at 30°C, and three cycles of 23 s in wax at 62°C.

## Procedure

**Figure 2:**
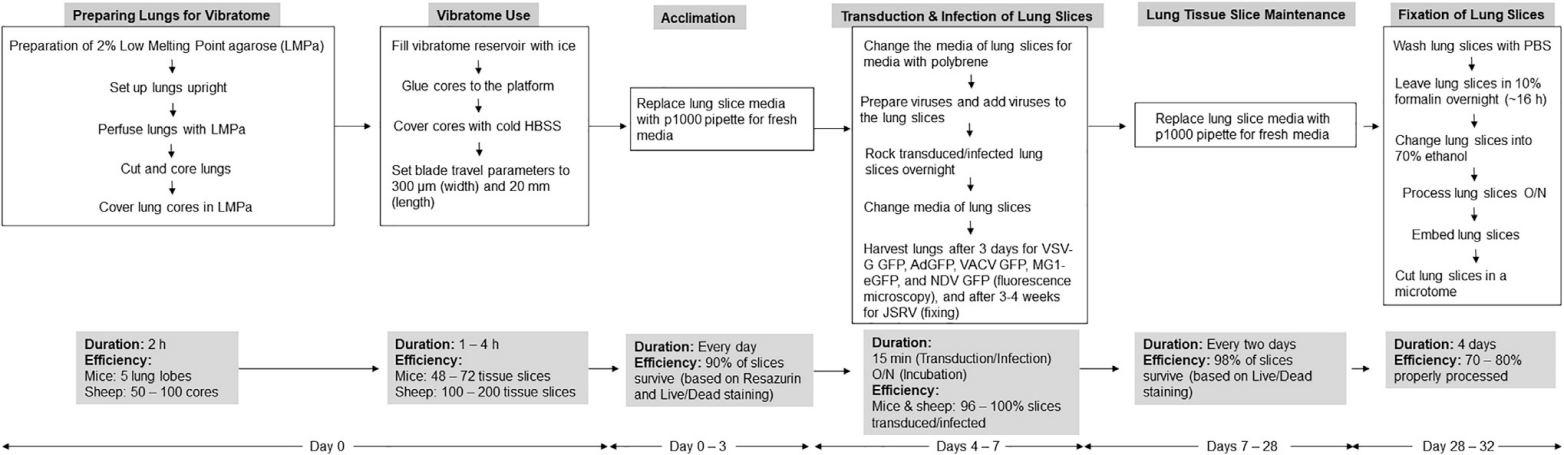
Experimental Overview and Timeline for Generating Precision-Cut Lung Tissue Slices for Viral Transduction or Infection

Please consult Figure 2 for an overview of the procedure. Note that the steps in this protocol should all be performed in a containment level or biological safety level 2 laboratory within a type II biological safety cabinet because the viruses used in this protocol are designated as risk group 2 pathogens and because the lungs should be kept as sterile as possible. All animal experiments were approved by the Institutional Animal Care Committee of the University of Guelph in accordance with Canadian Council on Animal Care (CCAC) guidelines.

1. Preparation of low melting point agarose (30 min) (day 0)

Prepare 2% LMPa in 1X HBSS 1 h prior to procuring lungs (see the Reagent Setup section). Depending on the lung size, the volume may range from 60 mL to 200 mL for lamb-derived lungs or less than 5 mL for all five lung lobes from a mouse.

2. Preparation of lung tissue for sectioning with a vibratome (2 hr) (day 0)

It is important to note that this step must be performed quickly, shortly after euthanasia of the animal, while the lungs are warm, and the agarose is still in its liquid state to ensure that it can completely perfuse the lung tissues. In the case of mice, remove the lungs, separate each lobe, and inject LMPa gel using a 5 mL syringe directly into each lobe by inserting the needle into the tissue and perfusing slowly and carefully so as not to damage the lung architecture. Stop once noticeable inflation is observed; this typically requires about 1 mL of LMPa per murine lung lobe.

In the case of sheep, set up the lungs as shown in Figures 1A and 1B. Set up two styrofoam box lids by covering them with paper towels and plastic (Saran) wrap. Spray them with 70% ethanol and prop them against each other with wooden skewers. Cut off the sheep trachea 2 inches from the primary bronchi and hold it in an upright position with a clamp. Use another clamp to secure the clamps already holding the trachea to the vertical styrofoam box. This will ensure the lungs remain upright throughout the entire procedure. Remove one of the 50 mL conical tubes containing LMPa gel from the 42°C water bath and set aside at room temperature to cool for 5 min to avoid burning the tissue. Then, using a 20 mL syringe filled with LMPa, inject the liquefied gel down the trachea into the lungs.

The rate of flow of the gel going down the trachea into the lungs should be relatively fast so as to avoid solidification of the LMPa due to overcooling (Table 2). Once there is an observable increase in lung volume as evidenced by a slight inflation in the lungs, it can be assumed that the lungs have been properly perfused (Figure 1B). Clamp the lungs shut and cool them by submerging them in cold (4°C) 1X HBSS on ice for 45 min to 1 hr for sheep and 30 min for mice to allow the LMPa to solidify.

**Table 2:** Troubleshooting Tips for Preparing Ovine and Murine Lung Tissue Microslices

Observation	Possible Explanation	Troubleshooting
Dried-up, leathery texture	lungs are getting colder	work fast; if LMPa gel does not go easily, then directly re-perfuse individual cores with LMPa gel^a^
Bloody, dark spots in lung lobes	lungs too hot time in hot room exceeded	do not pour LMPa gel; cut lung lobes and directly perfuse the cores instead^a^
LMPa gel rate too fast	agarose too hot	wait at least 1 hr after preparing the LMPa gel^a^
LMPa gel rate too slow	lungs are cold	ensure that the lungs are fresh so that core temperature remains warm if delayed, lungs can be left within bag inside styrofoam container at 37°C room for no more than 30 min directly perfuse the cores instead
Lungs appear mushy when cutting sections	agarose poured too hot waited less than 45 min for agarose to solidify	check that the conical tube containing LMPa gel feels warm and not hot to touch^a^ leave lungs incubating for longer to allow them to solidify
Blade is pushing the core	lungs not properly perfused blade is in contact with cartilage core-gel block not fastened to platform	re-infuse the core directly using warm LMPa gel with 5 mL syringe and a 30G needle have surgical scissors at hand to cut the cartilage glue core-gel block again or use another one
Tissue is sheared	agarose poured too hot lung tissue is over-perfused ice melting in reservoir	check that the conical tube containing LMPa gel feels warm and not hot to touch^a^ select another core according to Figure 2 criteria add more ice to reservoir; ensure that vibratome platform remains cold throughout procedure; drop a small ice cube on top of the tissue if possible.
Lung tissue media appear cloudy	not enough washes	wash the lung slices at least 5 times prior to culturing them and make sure to change the media with fresh media every other day

a Avoid if possible

Proceed to cut the ovine lungs with a scalpel into smaller 2–3-mm-thick sections (Figure 1C) and use an 8 mm biopunch to core each section (Figure 1D). The final core should be approximately 2 mm thick with an 8 mm diameter. For mice, each individual lung is the appropriate size and will not need to be cored.

Pour a thin layer of LMPa over a Petri dish that is on ice to provide a base to completely surround the cores with LMPa. Once this layer has solidified, transfer each sheep lung core or murine lung lobe into this Petri dish. Next, pour another thin layer of LMPa over them, leaving part of the core exposed; do not cover the sections completely yet. This will secure each core or lobe and prevent them from floating. Once this layer has solidified, pour enough LMPa to completely cover the cores or lobes (Figure 1E). Leave the Petri dish with the LMPa-embedded lung tissue on ice for 30 min to allow the agarose to completely solidify.

3. Sectioning with the vibratome (1–4 hr) (day 0)

**Figure 3:**
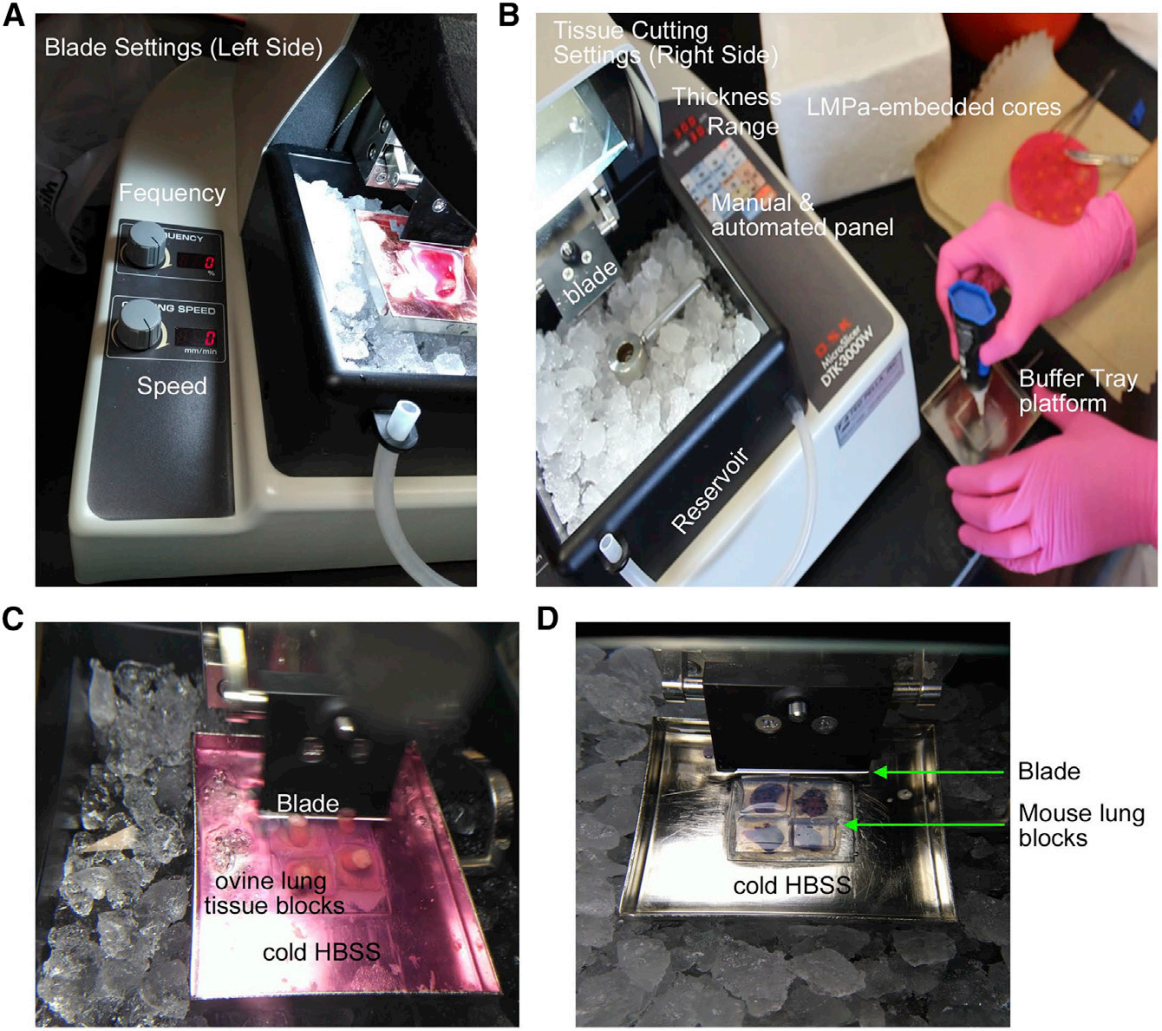
Equipment Setup for Use of the Vibratome The vibratome contains two setting panels: (A) a blade settings panel on the left and (B) a tissue cutting settings panel on the right. The blade settings panel can adjust the frequency and speed of the setting, while the tissue cutting settings accounts for the thickness of the slice and the blade travel range along the buffer tray platform. The reservoir should be filled with ice throughout the procedure. (C) and (D) show representative tissue blocks for ovine and murine lung tissues, respectively.

Fill the vibratome reservoir with ice. Replenish the ice to ensure the buffer tray platform remains cool at all times. Set the vibratome blade frequency to 100% and the speed to 8 μm/s using the blade settings panel (Figure 3A). Spread glue over the buffer tray platform (Figure 3B). Using a scalpel, cut out a block containing a core or lobe from the Petri dish with around 2 mm of agarose still surrounding the tissue, dab the block dry with a Kimwipe, and place the block on top of the glue to fasten the tissue block to the vibratome platform. We recommend fitting as many blocks side by side as possible to increase yield (e.g., 4 to 6 blocks can fit in the platform) (Figures 3C and 3D). Bring the surgical scissors to cut cartilage when necessary (Table 2).

Set the vibratome thickness parameter to 300 μm and adjust the blade travel range to cover the distance the blade will travel (40 mm should cover the platform) using the width and sectioning range (W/R) function in the control panel (Figure 3B). Once everything is set up, insert the ceramic blade into the blade holder and position the blade above and behind the blocks by selecting the Manual function and then pressing the Down button to lower the platform; press the reverse (Rev) button to place the blade behind the blocks. Begin cutting by selecting the Auto function and pressing the Start button; ensure that the blade is cutting and not simply pushing the block (Table 2) (please refer to Troubleshooting section). You may press Stop at any time.

Once the blade has sectioned through the tissue and a slice has been generated, remove the slice and place it in lung slice wash medium (LSWM) (Table 1). Once all lung slices have been collected, use forceps to place the lung slices in a 48-well plate (1 slice per well). To reduce the chances of contamination, wash each slice with 200 μL of LSWM five times using a p1000 pipette, incubate in 200 μL of the appropriate maintenance media, including antibiotics penicillin/streptomycin, gentamycin, amphotericin (refer to Table 1 for exact composition), and change the media every other day (Figure 1F). The slices will typically last up to 4 weeks in these conditions.

**Note:** Avoid suction with pasteur pipettes and a vacuum aspirator, which can readily aspirate the lung tissue slices, suctioning them right to the waste.

4. Acclimation period (days 0–3)

Maintain the lung slices by continuing to change the media for fresh media (200 μL) every day for 3 days prior to infection.

5. Viability measurement (days 3 and 28)

**Figure 4:**
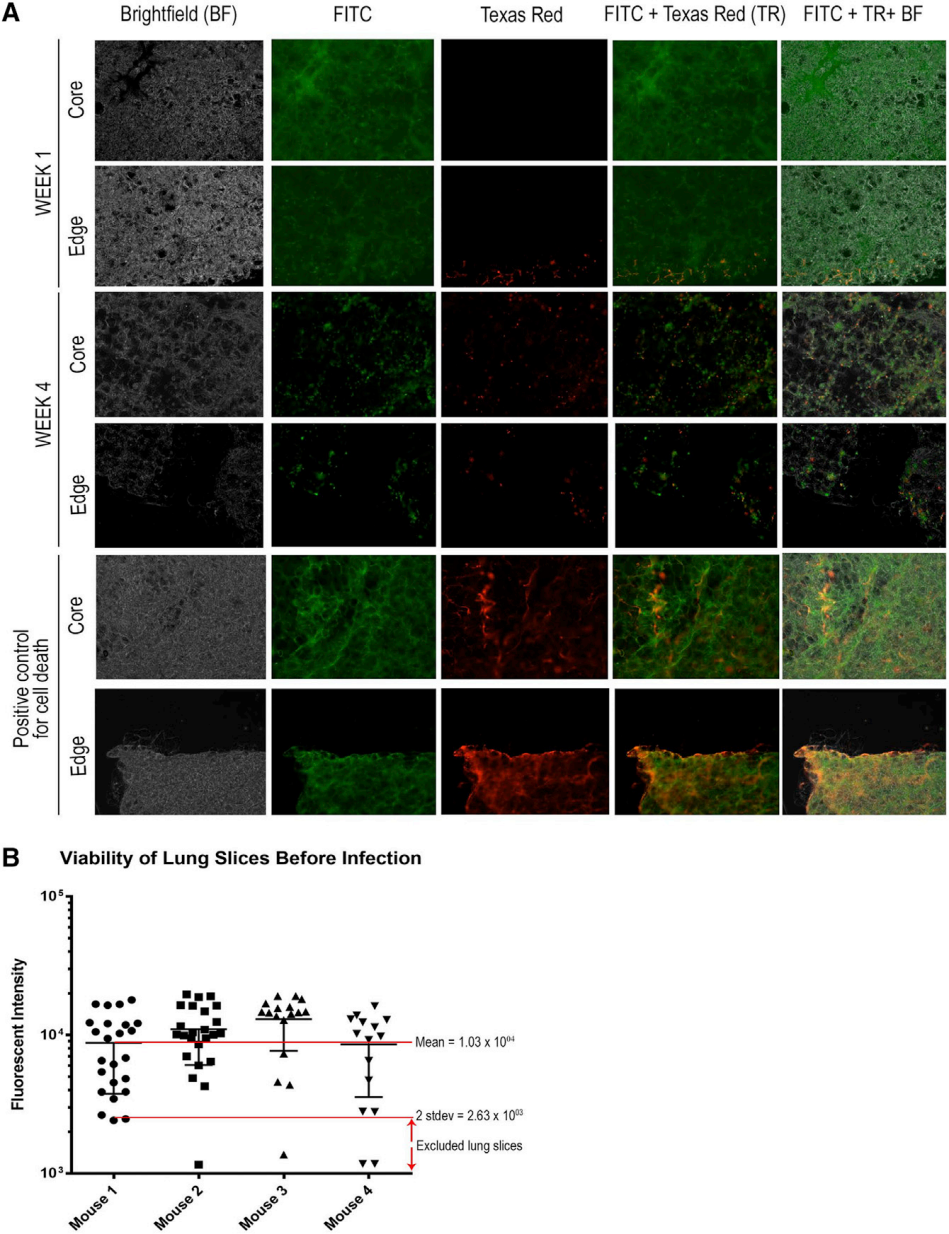
Sheep Lung Slices Remain Viable for a Month (A) Sheep lung slices were cultured in lung slice maintenance medium-C (Table 1) and harvested after 1 and 4 weeks. At both time points, the lung slices were tested for viability using a live-dead viability stain, and the results were visualized using a Zeiss Leica inverted fluorescence microscope using the FITC (for live cells) and Texas Red (for dead cells) channels. Lung slices treated with 10% Triton X-100 and left in the fridge for 24 hr were used as positive controls for cell death. After 4 weeks, slices where there was more observable red than green throughout the entire tissue slice were deemed as dead. (B) Mouse lung slice viability was assessed by a metabolic assay. Resazurin was added to mouse lung slices in LSMM-B for 2 hours and then the fluorescent intensity of each sample was read by a plate reader (530/25 nm; 595/5 nm). Lung slices 2 more SD below the mean were excluded from experimentation (indicated by red arrows). Error bars represent SEM.

The viability of slices can be assessed using a live-dead viability stain, with results visualized using an inverted fluorescent microscope (Figure 4A) or using a resazurin viability assay (Figure 4B), and can be done during the acclimation time window (day 3) to select viable slices for infection and during and after infection (day 28). Live-dead staining has been previously detailed.^31^ Briefly, the stain is composed of two dyes: (1) a green dye that easily transverses the cell membrane and (2) a red dye that can only enter cells if there is damage to the cell membrane. Remove the media from the lung slices with a p1000 pipette and wash twice with 1X PBS before adding the stain. Lung slices can be treated with 10% Triton-X and left in the fridge for 24 hr, to be used as a positive control for cell death, before being treated with the stain. Visualize lung slices with a fluorescence microscope according to manufacturer specifications.

The resazurin assay has also been described before.^32^ Add LSMM-B containing resazurin to lung slices in 48-well plates. Incubate plates in a 37°C, 5% CO_2_ incubator. After 2 hr, move each lung slice into a 96-well plate with fresh LSMM-B without resazurin for quantification of fluorescence using a plate reader. Resazurin is converted to a fluorescent compound by metabolically active cells, and, therefore, higher fluorescent values correlate with greater viability. We arbitrarily defined lung slices as unviable when they had fluorescence values equal to or lower than 1 SD below the mean of untreated lung slices and recommend excluding those from experiments (Figure 4). Typically, 93%–99% of slices remain viable after the procedure (Figures 1F and 4) when using the maintenance medium recipe detailed in this paper.

6. Transduction and infection of lung slices (15 min) (days 3–4)

Production of JSRV, LVs, Ad-GFP, MG1-eGFP, VACV-GFP, and NDV-GFP has been extensively described in the literature,^31^, ^33^, ^34^, ^35^, ^36^, ^37^, ^38^, ^39^, ^40^, ^41^ and we have used similar methods. For retroviruses and lentivectors, remove media from the lung slices and add fresh media containing polybrene at a final concentration of 8 μg/mL. Next, infect with 2 × 10^6^ U (reverse transcriptase units) of JSRV concentrated by ultracentrifugation or 1 × 10^6^ transducing units (TU)/mL for the VSV-G GFP LV. For the other viruses, polybrene is not needed; simply replace with fresh media and add 1 × 10^8^ plaque forming units (PFU)/mL for MG1-eGFP, 5 × 10^6^ PFU/mL for VACV-GFP, 1 × 10^7^ PFU for NDV-GFP, or 1 × 10^7^ PFU Ad-GFP per lung slice dropwise. Place the 48-well plate on a rotating platform (nutating mixer fixed speed 120 V) inside a tissue culture incubator at 37°C with 5% CO_2_ overnight. The following morning, remove media containing input virus from lung slices, wash five times with PBS to remove residual input virus from the agarose impregnated tissue, and add fresh media back to the lung slices.

7. Detection of viral transduction and infection (days 5–7)

**Figure 5:**
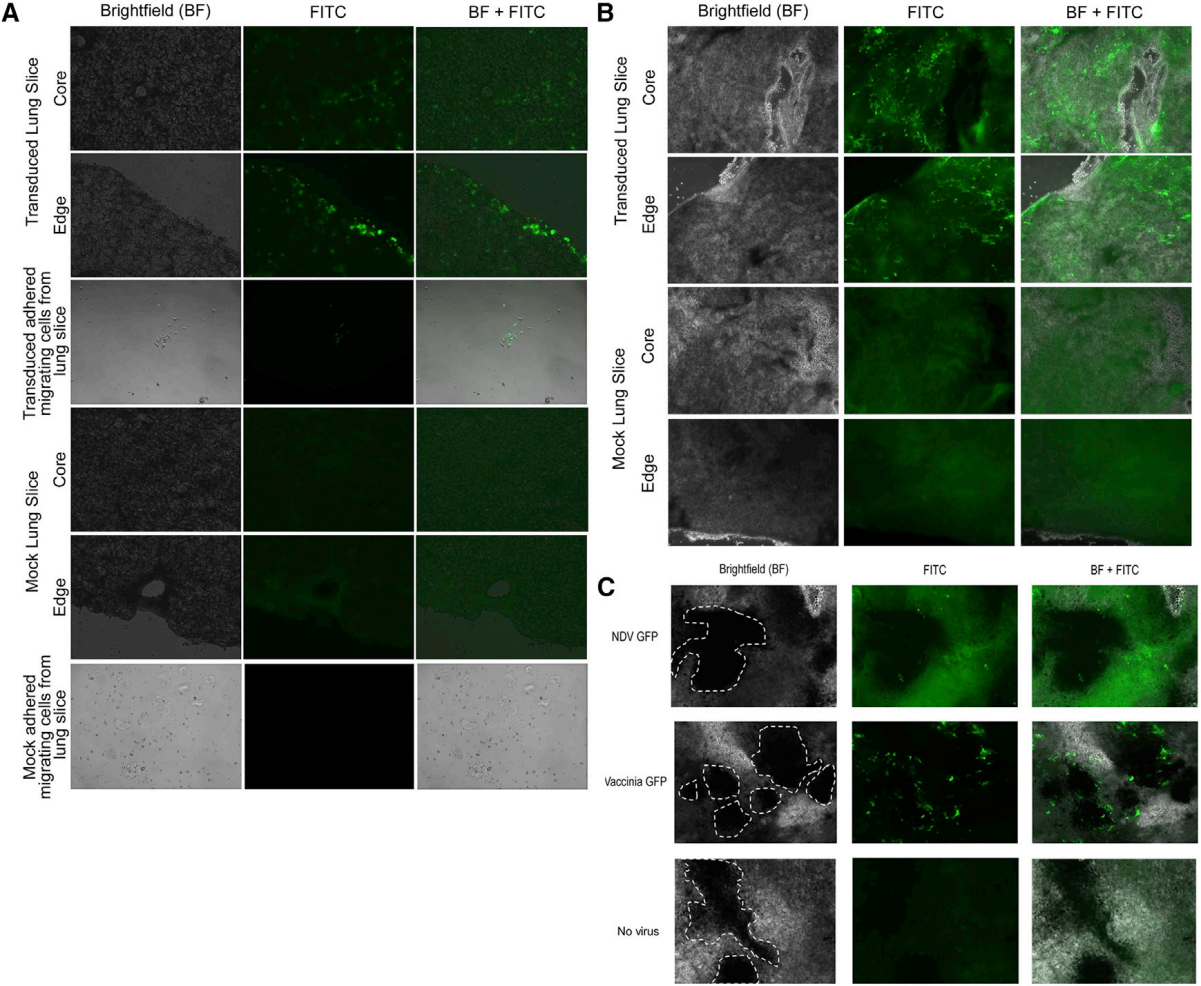
Ovine and Murine Lung Slices Can Be Transduced with Gene Therapy Vectors and Murine Tumor-Bearing Lung Tissue Slices Can Be Infected with Oncolytic Viruses VSV-G-LV-transduced ovine lung cells (A) and Ad-GFP-transduced mouse lung cells (B) can be seen as green fluorescent foci in the center and edges of the lung slices. Transduction was possible in standard cell culture medium supplemented with additional antibiotics (LSMM-A; Table 1). Migrant cells from the lung slices are also susceptible to transduction (A). Lung slices were imaged by fluorescence microscopy to detect infection with NDV-GFP or VACV-GFP 72 hr post-infection (C). B16-F10 tumors are outlined in white dotted lines in the BF images. GFP expression can be observed as punctate spots in and around the tumors. Uninfected lung slices show dark B16-F10 tumors (arrows). GFP puncta localized in and/or around the tumors can be identified in NDV-GFP- and VACV-GFP-treated slices.

**Figure 6:**
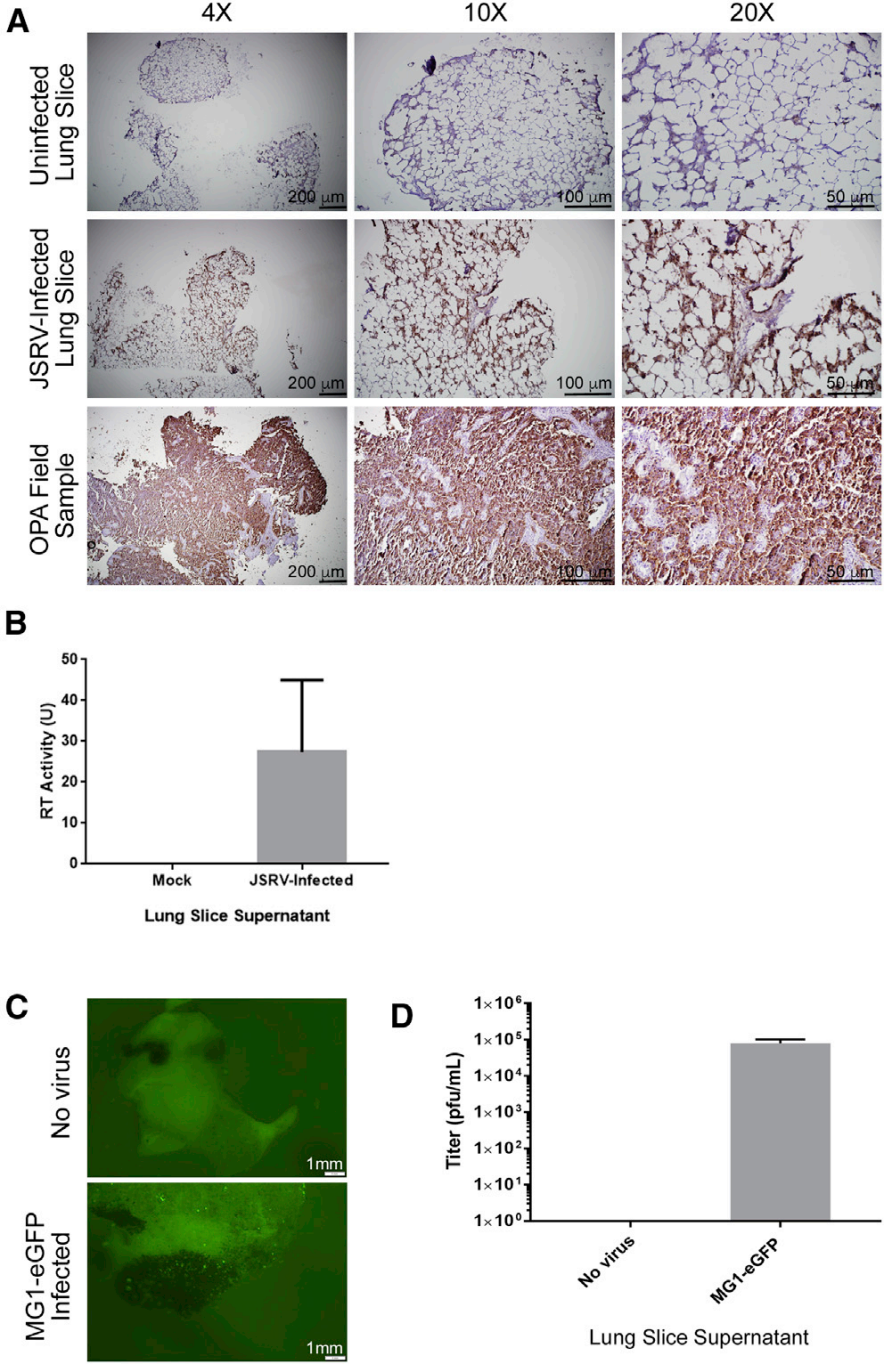
Sheep and Murine Lung Slices Can Be Infected and Virus Production Can Be Detected Lung slices grown in LSMM-C were infected with JSRV and incubated for 4 weeks at 37°C. (A) Robust immunohistochemical staining of a lung slice infected with JSRV using a monoclonal antibody against the JSRV Env oncoprotein. An uninfected lung slice was used as a negative control and lacked staining. Tissue from the lung of an adult sheep suffering from ovine pulmonary adenocarcinoma was used as a positive control. (B) Reverse transcriptase (RT) activity in the supernatant of mock and JSRV-infected lung slices. Murine B16-F10 melanoma tumor-bearing lung slices grown in LSMM-B were infected with oncolytic Maraba virus (MG1-eGFP). (C) Top: uninfected lung slices show dark B16-F10 melanoma tumors (arrows). Bottom: GFP puncta localized around and within the tumor (arrows) indicates infection by MG1-eGFP. (D) MG1-eGFP virus is detectable in supernatant from infected lung slices. Viral titers (1 × 10^5^ PFU/mL) from infected lung slice supernatants (N = 6) were determined by TCID50 48 hr post-infection. Error bars represent SEM.

At 48–72 hr post-transduction, visualize the lung slices transduced with VSV-G GFP LV (48 hr) (Figure 5A) or infected with Ad-GFP (72 hr) (Figure 5B), VACV-GFP (72 hr), NDV-GFP (72 hr) (Figure 5C), or MG1-eGFP (48 hr) (Figures 6C and 6D) using a fluorescent microscope. For JSRV, continue to maintain the lung slices as described previously for 3–4 weeks prior to fixing and performing immunohistochemistry (IHC) (Figures 6A and 6B). Collect media from lung slices infected with JSRV to measure reverse transcriptase activity as described previously.^42^

8. IHC (4 days) (weeks 3–4)

Remove media from the lung slices and wash them twice with 1X PBS. Fix the lung slices in 10% formalin for approximately 16 hr. After fixation, wash the lung slices three times with 1X PBS before placing them in 70% ethanol. Transfer the lung slices to an embedding cassette by spreading them on top of two foam pads and then store in 70% ethanol. Place the cassettes in an automated tissue processor (ThermoScientific Excelsior ES) and begin fixation, dehydration, and paraffin wax infiltration of the tissues by following the program described in the Equipment Setup section of this paper.

**CAUTION:** Do not use conventional overnight processing programs, as this might cook and shear the slices.

Once finished, proceed to embedding tissues in paraffin blocks using an embedding work station (ThermoScientific HistoStar) and protocols as described in the instruction manual. The lung slice paraffin blocks should be stored at −20°C, since blocks should be cold during sectioning. The next day, use a microtome (ThermoScientific Finesse ME+) to prepare 5 μm sections from the blocks. Keep blocks on ice while waiting to use the microtome.

**CAUTION:** Do not use the trim function; instead, use “section” or you risk losing the lung slices.

Using tweezers, place tissue sections in a 42°C water bath and, using clean tweezers, place the section onto a Superfrost Plus slide. Charged slides improve tissue adhesion to the glass. Leave the slide on a heat block at 37°C to dry overnight (>16 hr). The next morning, proceed to IHC as previously described.^31^, ^43^

### Timing

• Day 0: prepare tissue for vibratome and slice tissue. Wash slices.
• Days 0-3: maintain and culture lung tissue slices in appropriate maintenance medium.
• Days 3–4: infect and transduce lung slices.
• Day 5: change media of lung slices.
• Days 5–7: check for fluorescence of GFP reporter gene. Take pictures. Collect media from slices to measure virus titer.
• Weeks 2–3: continue culturing lung slices for JSRV. Collect media from lung slices infected with JSRV to measure reverse transcriptase activity.

For long-term experiments:

• Day 28: collect lung slices and fix for 16 hr in 10% formalin.
• Day 29: place lung slices in cassettes and store in 70% ethanol overnight and proceed to tissue processing.
• Day 30: paraffin-embed lung slices into blocks and leave in freezer overnight.
• Day 31: cut specimen blocks, mount on slides, and leave baking overnight.
• Day 32: perform IHC to visualize virus transduction.

### Troubleshooting

#### Tissue Preparation and Cutting

Please consult Table 2 for troubleshooting during tissue preparation and vibratome use.

#### Lung Slice Architecture

Although sheep lung tissue slices can be grown for up to 4 weeks in culture, there is a reduction in size over time (Figure 6A). This loss of structure could be due to the dissolution of agarose.^1^ To reduce this effect, we recommend making wider slices (>8 mm) for longer experiments.

#### Variability in Lung Slice Viability

We recommend generating large numbers of tissue slices from each murine lung sample to obtain a more representative mean fluorescence for the resazurin assay.

#### Potential Pitfalls during Tissue Preparation

This protocol requires an uninterrupted day and depends on the experimenter’s speed and efficiency. We recommend obtaining lung slices that are as fresh as possible. The shorter the time the lungs are outside the body prior to perfusion with LMPa gel, the better the quality of the lung slices (Table 2). In general, PCLSs range in size between 150 μm to 500 μm, although most studies use sizes between 250 μm and 300 μm.^1^, ^2^, ^3^, ^4^, ^6^, ^7^, ^8^, ^11^, ^12^

With respect to the vibratome, two main parameters need to be optimized: (1) the frequency of vibration to create an easier cutting path for the blade and (2) the speed of the blade to generate slices in a timely manner; these parameters are specific to each type of tissue. The recommended manufacturer frequency and speed settings for tissues such as lung are 80%–100% and 10 μm/s in the case of the vibratome we used. In this study, we preferred using 100% and 8 μm/s for frequency and speed, respectively, for lung tissue slices. With these settings, we were able to generate lung tissue slices at a rate of 1 slice/min. Though it was possible with certain cores to increase the speed, prolonged speed increments had the potential of warping and shearing lung tissue slices. In fact, with a speed greater than 10 μm/s, the blade would move the block off the platform instead of cutting the tissue.

The vibratome procedure can take between 1 to 4 hr, depending on how well the tissue is being cut. The vibratome used in this study comes equipped with a very sharp ceramic blade; however, we found that cutting heavily depends on the tissue architecture. The amount of time spent with cutting depends on the variation in perfusion and the presence of cartilage, which sometimes needs to be removed. We recommend adding up to 6 blocks into the buffer tray platform to yield as many lung slices per minute as possible and decrease the time it takes to complete the procedure. We also suggest keeping a watchful eye during the entire procedure to ensure that: (1) the blade is not pushing the tissue; (2) cartilage is not preventing efficient cutting; and (3) the platform is not heating up. The frequency of the vibration can introduce heat. Adding ice to the platform and the buffer tray platform can ensure consistently low temperatures.

#### Lung Tissue Slices Preparation Time

To shorten tissue preparation times, we considered freezing tissues post-LMPa perfusion; however, freezing at this point significantly reduced tissue viability (data not shown). Freezing tissues after cutting with the vibratome, however, did preserve viability. In this case, tissues were frozen in 10% DMSO-LSMM-C (Table 1) after 2 days of culturing. Upon addition of the freezing media, the lungs were frozen overnight at −80°C before being transferred to a liquid nitrogen tank for long-term storage. Thawing was rapidly executed at 37°C, and the slices were allowed to culture for 3 days to 1 week prior to experimental use. Although thawed slices did not seem to have reduced viability, freezing drastically reduced transduction efficiency. Therefore, we do not recommend freezing for lung slice work that involves viral infections or transductions. Recent studies have looked at cryopreservation of lung slices and have also noted similar drawbacks.^2^, ^6^ Perhaps a longer acclimation period could resolve this problem.

#### Transduction and Infection of Lung Slices

After generating the slices with the vibratome, we recommend acclimating the lung tissue slices in culture for 3 days prior to transduction or infection with the lung slices. We found that this acclimation period was important for transduction and infection efficiency. We also found that transducing with GFP-expressing viruses yielded punctate foci indicative of efficient transduction of the lung slices (Figures 5A–5C). However, it is important to note that a strong promoter should be used to drive expression of the GFP reporter gene. Lung slices grown for more than 3 weeks exhibited large amounts of auto-fluorescence that were difficult to discern from positively transduced foci, compared to negative untreated tissue. Other studies have also noted auto-fluorescence in the lungs as a potential drawback from using GFP as a reporter gene.^44^, ^45^ Therefore, we recommend using other reporter genes, such as mCherry and heat stable human placental alkaline phosphatase (hPLAP), for long-term studies.

However, we found that, for sheep lung slices from lambs less than 6 months of age, the hPLAP reporter gene is not a viable option due to the fact that lambs express a form of heat-stable AP in their lungs (Figure S1). Therefore, other reporter genes, such as mCherry, nuclear β-galactosidase, or firefly luciferase, might be useful for ovine lung tissues. Alternatively, the use of IHC could circumvent the autofluorescence problems observed. We were able to detect the presence of GFP using a rabbit anti-GFP (Invitrogen, A11122), while no GFP was detected in the no virus control (Figure S2). Furthermore, using murine lung tissue slices that naturally do not express heat-stable AP, we were able to effectively transduce these lung tissue slices with VSVg hPLAP LV, and EBOV G-pseudotyped hPLAP LV (Figure S2).

### Anticipated Results

The use of tissue slices is gaining widespread use^1^, ^2^, ^4^, ^5^, ^6^, ^8^, ^9^, ^10^, ^11^, ^12^ and becoming an attractive alternative to *in vitro* cell culture experiments due to the presence of a cellular framework and microenvironment similar to that observed *in vivo*.^1^, ^2^, ^4^, ^5^ In this study, we optimized the maintenance of ovine and murine lung tissue slices in culture to evaluate small ruminant betaretroviruses (JSRV), gene therapy vectors VSV-G GFP LV and Ad-GFP, and OVs MG1-eGFP, VACV-GFP, and NDV-GFP. With this protocol, we were capable of transducing and maintaining infection with all of the aforementioned viruses in lung slices (Figures 5 and 6). The media recipes we used to maintain infection with these viruses (Table 1) can serve as a reference for future virus work in organotypic slices but might need to be optimized, depending on the transcription factors important for the virus. For instance, JSRV’s promoter region has two lung and liver-specific hepatocyte transcription factor (HNF-3) binding sites,^46^, ^47^, ^48^, ^49^, ^50^, ^51^ and we found that addition of these two transcription factors was better for infection. We also measured viability using two different assays: a live-dead stain for ovine lung slices and a well-established resazurin assay for murine lung tumor slices.^31^, ^52^ Using the live-dead viability assay, we were able to determine that the sheep lung slice tissue could remain viable for approximately 1 month (Figure 4A), longer than other studies, regardless of the species and tissue type.^8^, ^9^, ^12^, ^19^, ^31^ Interestingly, we found that the sheep lung tissue slices that were viable and capable of harboring transduction or infection (Figures 5A and 6A) would often be accompanied by migrating lung fibroblast-like cells that would adhere to the bottom of the plate. We ascertained that this observed characteristic was a marker of tissue slice viability. Interestingly, these adherent cells also had the capacity of being transduced by the VSV-G GFP LV (Figure 6A). These cells could be harvested as a primary cell line and constitute another reason for the use of tissue slices. We also took advantage of the resazurin assay to determine murine lung slice viability without compromising the ability to subsequently infect them with viruses. Resazurin can be added to lung slice media for 2 hours and then washed and replaced with normal media without significantly affecting lung slice viability. Thus, this protocol provides two alternative methods for measuring viability that future studies could benefit from. We also found that the OVs were mostly found in tumor tissues or the peri-tumoral region in the lung slices (Figures 5C and 6C), as has also been well established in the literature.^53^, ^54^, ^55^, ^56^, ^57^, ^58^ Moreover, we observed a decrease in tumor tissue metabolic activity when in the presence of oncolytic vector MG1-eGFP, using the resazurin assay, a surrogate tool for viability (Figure S3). Therefore, this protocol is a convenient tool to assess the activity of other gene and OV vectors prior to and to complement *in vivo* work. We expect that this protocol can also be applied to other pathogens and other tissues.

## Author Contributions

M.C.R.G. developed the protocol, produced JSRV and VSV-G GFP LV, generated and maintained all lung slices, and measured the viability of lung slices with the live-dead stain. J.P.vV. delivered B16-F10 melanoma cells to the mice, propagated MG1-eGFP and VACV-GFP, infected lung slices with MG1-eGFP, VACV-GFP, Ad-GFP, and NDV-GFP, and measured the viability of the lung slices with resazurin assays. L.A.S. generated the NDV-GFP construct and propagated the virus. J.dJ. and R.C.M. propagated the Ad-GFP virus. VACV-GFP construct was generated by A.P. and was generously gifted by J.C.B. M.C.R.G. and J.P.vV. wrote the manuscript with the help of L.A.S., R.C.M., A.P., J.C.B., B.W.B., and S.K.W.

## Conflicts of Interest

The authors declare no competing financial interests.

## Supplemental Information

Document S1. Figures S1–S3

Document S2. Article plus Supplemental Information
