# Supplementary material for: Use of Precision-Cut Lung Slices as an Ex Vivo Tool for Evaluating Viruses and Viral Vectors for Gene and Oncolytic Therapy
Source: Mol Ther Methods Clin Dev. 2018 Aug 4;10:245–56. doi: 10.1016/j.omtm.2018.07.010 (PMC6092314; doi:10.1016/j.omtm.2018.07.010)
Supplement: Document S1. Figures S1–S3 [file mmc1.pdf]

OMTM, Volume *10*

Supplemental Information

Use of Precision-Cut Lung Slices as an *Ex Vivo* Tool for Evaluating Viruses and Viral Vectors for Gene and Oncolytic Therapy

María C. Rosales Gerpe, Jacob P. van Vloten, Lisa A. Santry, Jondavid de Jong, Robert C. Mould, Adrian Pelin, John C. Bell, Byram W. Bridle, and Sarah K. Wootton

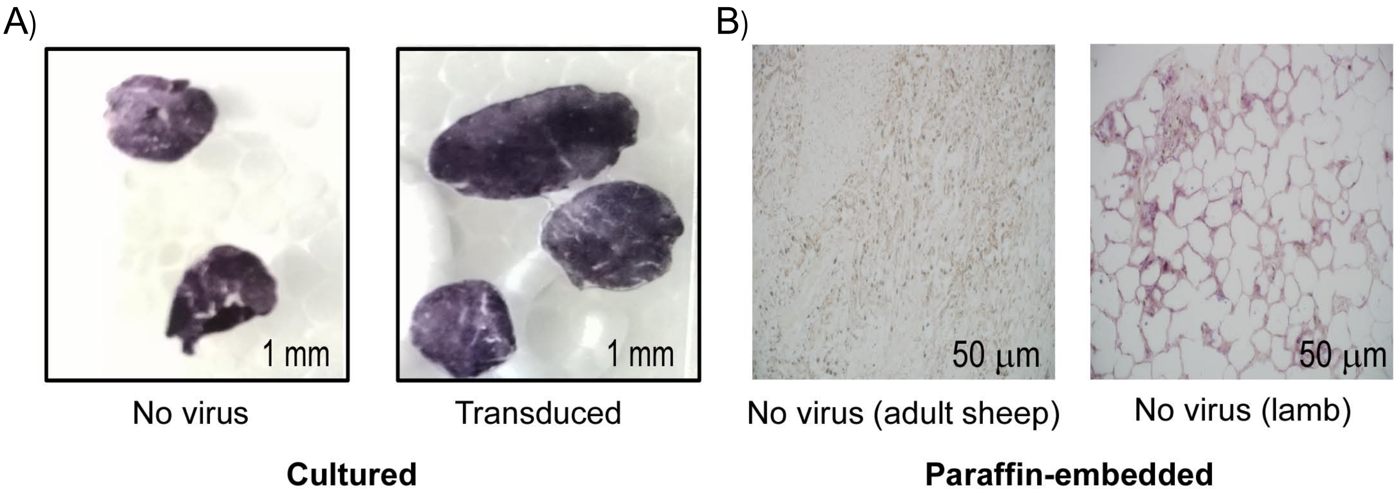

Supplementary Figure S1. Unlike adult ovine tissue, young ovine tissue (lambs less than 6 months of age) expresses a heat stable alkaline phosphatase (AP). (A) Ovine lung tissue slices from a 4-month old lamb shows staining for AP post heat-inactivation at 65˚C for 1 h suggesting presence of heat stable AP. (B) In contrast to young lamb tissue, heat-inactivated paraffin-embedded tissue from adult sheep (left) does not show purple staining characteristic of AP (right).

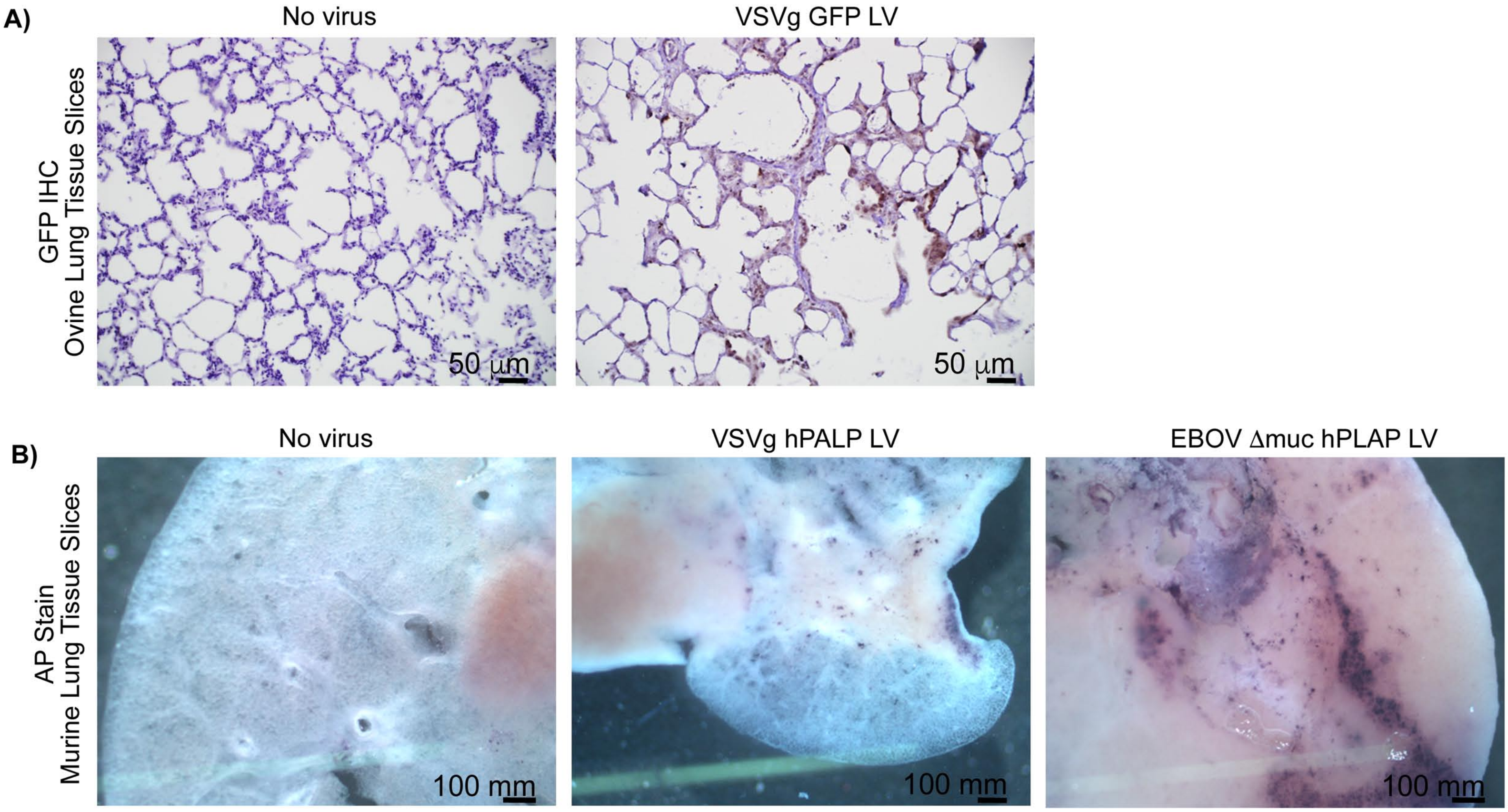

Supplementary Figure S2. Immunohistochemical staining for GFP expression and enzymatic analysis of alkaline phosphatase expression as alternative reporter genes and detection methods to circumvent autofluorescence in the lung. (A) Immunohistochemical staining for GFP expression in ovine lung tissue slices not transduced (left) or transduced (right) with GFP-expressing VSVg pseudotyped lentivector. Transduced lung tissue cells expressing the GFP reporter gene are evidenced by stained brown. (B) Alkaline phosphatase (AP) staining of murine lung tissue slices not transduced (left) or transduced with VSVg (middle) and Ebola virus (EBOV) glycoprotein (right) pseudotyped lentivectors expressing the human placental AP reporter gene. Purple foci denote evidence of AP expression.

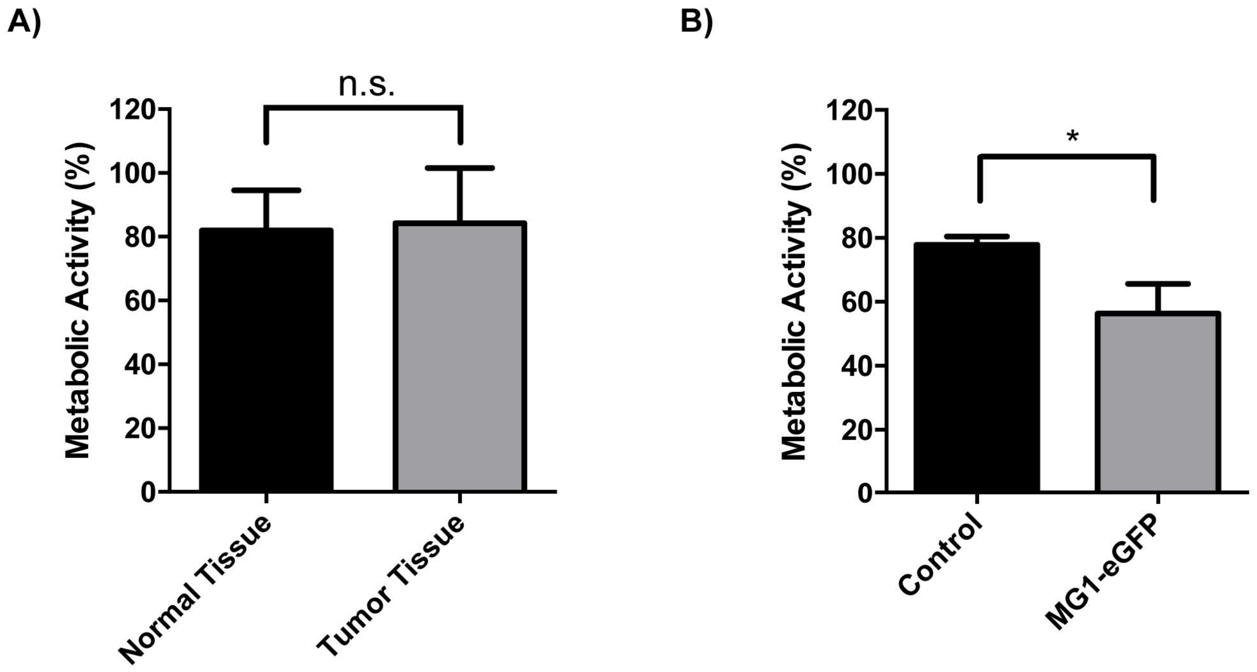

Supplementary Figure S3. Decreased metabolic activity in murine lung tumor tissue infected with oncolytic Maraba virus, MG1-eGFP. Cell viability, as measured using the resazurin metabolic assay, of normal and tumor bearing lung tissue from mice two hours prior to infection (A) and then again 48 h post-infection (B) with MG1-eGFP. Unpaired t-tests were conducted for each data set (A and B) using GraphPad Prism 7 software (GraphPad Software, LaJolla, CA, USA). The p values were 0.7751 (A) and 0.0124 (B).
